# A retrospective evaluation of bites at risk of rabies transmission across 7 years: The need to improve surveillance and reporting systems for rabies elimination

**DOI:** 10.1371/journal.pone.0197996

**Published:** 2018-07-02

**Authors:** Pasquale De Nardo, Elisa Gentilotti, Francesco Vairo, Boniface Nguhuni, Zainab Chaula, Emanuele Nicastri, Abbas Ismail, Giuseppe Ippolito

**Affiliations:** 1 Resource Centre for Infectious Diseases, Department of Internal Medicine, Dodoma Regional Referral Hospital, Dodoma, Tanzania; 2 ‘Lazzaro Spallanzani’ National Institute for Infectious Diseases INMI-IRCCS, Rome, Italy; 3 Department of Infectious Diseases, Tor Vergata University Hospital, Rome, Italy; 4 University of Dodoma - UDOM, Department of Statistics, Dodoma, Tanzania; University of Pretoria, SOUTH AFRICA

## Abstract

The vast majority of rabies deaths occur in developing countries and rural areas. Due to the absence of surveillance and the lack of reliable information, many endemic countries are not able to assess their rabies burden and implement appropriate solutions. This study reports the incidence of animal bites considered at risk of rabies transmission, along with rates and determinants of the adherence to post-exposure prophylaxis (PEP) between 2008 and 2014 in Dodoma Region, Tanzania. A retrospective analysis of rabid animal bites considered at risk of rabies transmission at Dodoma Regional Referral Hospital (DRRH) during 2008–2014 was conducted. Data were collected from the registers of patients presenting to the hospital because of a potential rabies exposure. The patients were assessed by a trained health worker and each bite was considered as “at risk of rabies” based on the victim’s description of the event. Overall, 10,771 patients coming from Dodoma Region attended DRRH because of a bite from a suspected rabid animal, giving a mean incidence of 74 bites at risk of rabies transmission per 100,000 persons per year. Overall, only 46.0% of people exposed received a complete course of PEP and 61.6% attended the clinic within 48 hours after the bite. Multivariate analysis shows that people age >15 years, residence in rural areas and occurrence during the rainy season were independently associated to delayed access to care. Male gender, age below 15 years. and bites occurring during the dry season were associated with completion of PEP. In this area with a high rate of at-risk bites, several factors—mainly related to health care access and to the affordability and delivery of rabies vaccines—still need to be addressed in order to reduce gender and social inequalities in rabies prevention and control. Further efforts are required to establish an efficient rabies surveillance system in Dodoma Region.

## Introduction

Rabies is one of the earliest infection recorded. Descriptions compatible with rabies were found in ancient text from Egypt, Persia and China [1]. Moreover, rabies is the infectious disease with the highest case-fatality ratio; once clinical signs appear, the disease is almost unavoidably fatal [2]. Despite the existence of post-exposure vaccines for victims of rabid-animal bites since 1885, an average 59,000 people die worldwide of rabies each year [3], the vast majority of these deaths occurring in Asia and Africa with little changes in its global distribution [4]. This deadly disease affects especially poor and vulnerable populations in remote rural communities with limited access to human vaccine and specific immunoglobulins [4].

In Tanzania, canine rabies is endemic, with more than 40,000 dog bites reported for the year 2000 and around 1500 human rabies deaths estimated to occur annually, giving a nationwide annual incidence of around 5 cases cases/100,000 [5]. However, the extent of the problem may be heavily underestimated [4]. Although all age groups are susceptible, rabies is most common in children younger than 15 years. In the north-western part of Tanzania, the incidence of rabies was 5 times higher in children under 15 than in adults [3,4].

Rabies is described as being “*100 percent fatal*, *100 percent preventable*” [6]. Rabies prevention and control can be achieved through the implementation of vaccine coverage among the dogs [6]. To effectively break the transmission cycle, approximately 70.0% of the local dog population needs to be vaccinated [7].

To prevent the risk of developing rabies in humans, the WHO recommends immediate treatment of the bite-victim through Post-Exposure Prophylaxis (PEP) measures depending on the type of contact with the suspected rabid animal [8]. For the categories at risk (category II and III), washing and flushing of all bite wounds and scratches for about 15 minutes with soap or detergent and copious amounts of water should be done as early as possible [8]. Individuals with WHO category II or III exposures should receive PEP without delay as an emergency procedure. Currently, the recommended WHO option is intradermal PEP regimen administered on days 0, 3 and 7. Intramuscular regimens are still considered valid options to be administered as follows: 1-site vaccine administration on days 0, 3, 7 and the fourth dose between days 14 to 28; 2-site vaccine administration on day 0 and 1-site on days 7 and 21 [8]. RIG should be administered for severe category III exposures. The availability and accessibility of PEP is extremely limited in most of Sub-Saharan Africa [9]. In particular, national studies show that official reports may underestimate rabies incidence by more than 100-fold, because most deaths occur in communities rather than in hospitals [7,9], and those occurring in hospitals are frequently misdiagnosed as encephalitis of unknown origin [10]. Generating accurate data on rabies incidence will be key to achieving control and elimination of this neglected disease in endemic areas [11].

We conducted a retrospective study to determine the incidence of humans being bitten by suspected rabid animals, the rate of adherence to the PEP and its determinants over the period 2008–2014 in Dodoma Region, Tanzania.

## Materials and methods

### Study site and procedures for rabies exposure management at DRRH

The study was conducted at Dodoma Regional Referral Hospital (DRRH), serving a population of 2,083,588. Data were retrospectively collected from the registers of patients attending the health facility because of a potential rabies exposure between 2008 and 2014. The risk assessment of developing rabies after an exposure was performed by trained health personnel. The WHO approach—based on epidemiology, characteristics of exposure and clinical criteria—was adopted during the study period [8]. The following exposure modalities were considered at risk of rabies transmission, thus requiring PEP: “nibbling of uncovered skin, minor scratches or abrasions without bleeding, licks on broken skin” (category II) and “single or multiple transdermal bites or scratches, contamination of mucous membrane with saliva from licks; exposure to bat bites or scratches” (category III). The Anti-Rabies Vaccine (ARV) regimen offered during the reporting period consisted in multiple intramuscular administrations of the human diploid-cell vaccine (H-DCV, Rabivax^®^) at day 0, 7, and 28. This schedule is not included among those recommended by WHO. Rabies immunoglobulin was not available at DRRH in 2008–2014.

### Data collection and statistical analysis

**Fig 1:**
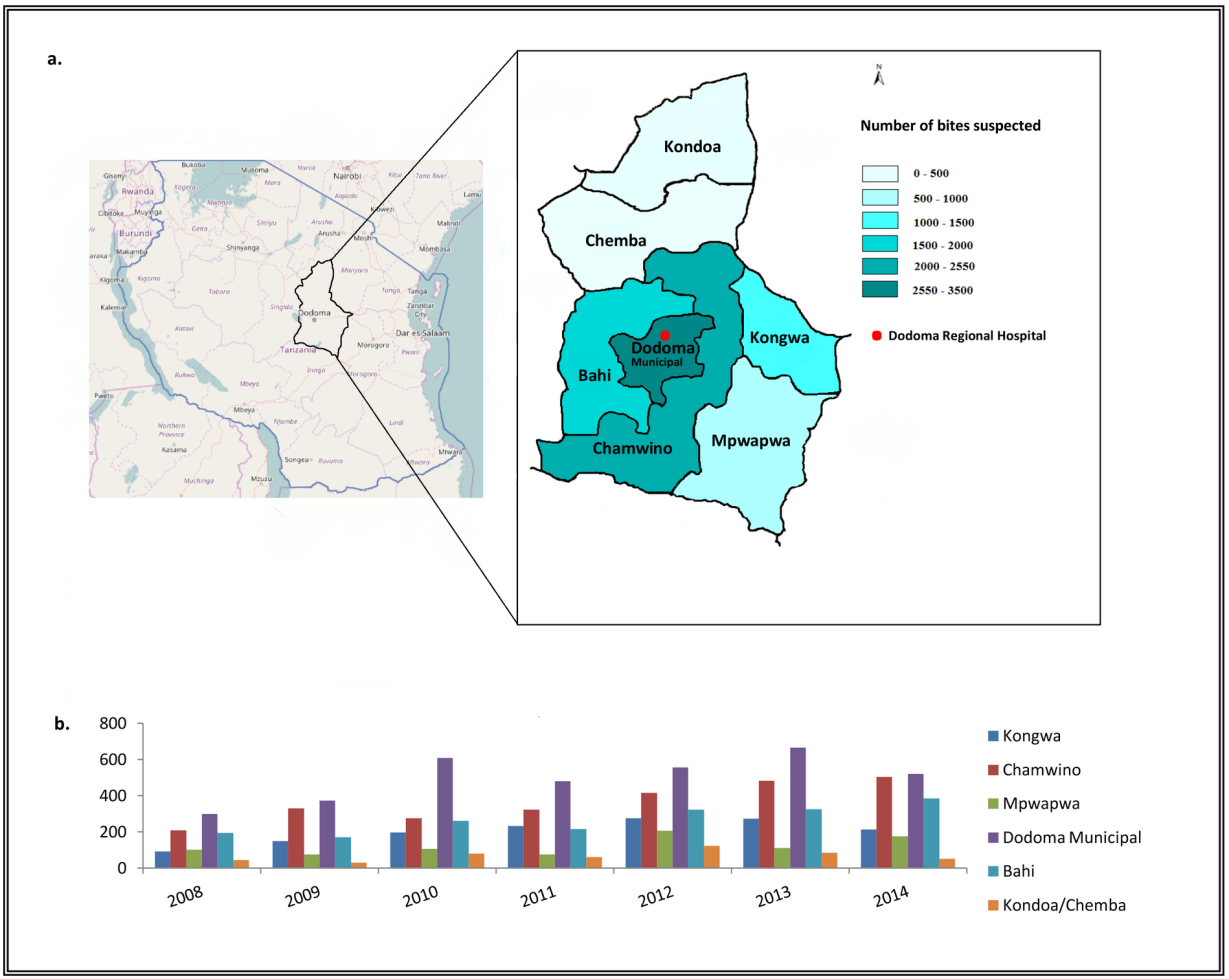
Total number of suspected rabid animal bites in Dodoma region. (a) Total number of suspected rabid animal bites from 2008 to 2014 distributed per district. (b) Diagram of number of suspected rabid animal bites per district and year.

A dataset including only the patients coming from Dodoma Region and considered at risk of developing rabies was created. The statistical analysis was conducted on this dataset. Information collected from the registers included patients’ age, sex and geographical location (district), the month and year of bite occurrence, the delay in attending the hospital categorized in ≤2 days, 3–7 days and >7 days of delay, the species of the biting animal, and the number of vaccine doses administered to the patient. All the districts outside Dodoma municipality were classified as “rural area”. Adherence to ARV and rabies awareness were calculated as number of doses received and time between the bite and hospital attendance (delay), respectively. Data were aggregated by districts, years, age, and sex. Incidence was calculated using the population estimates from the 2012 population and housing census [12]. Descriptive, univariate and multivariate logistic regression analyses were performed using SAS software version 9.3 [SAS Institute, Inc., Cary, NC, USA] with the aim to investigate the predictors of delay in attending health care and the variables associated with complete PEP intake (defined as receipt of all three doses, according to the regimen offered at DRRH) among individuals exposed. Statistical significance was assessed at α = 0.05. Tanzania map showed in Fig 1 was created using Landsatlook, available at https://landsatlook.usgs.gov/. Dodoma region map showed in Fig 1 was created by using Paintbrush (Microsoft) and Adobe Photoshop (Adobe Systems Incorporated).

The study received approval from the Dodoma Regional Health Management Team. All data analyzed were anonymized.

## Results

**Fig 2:**
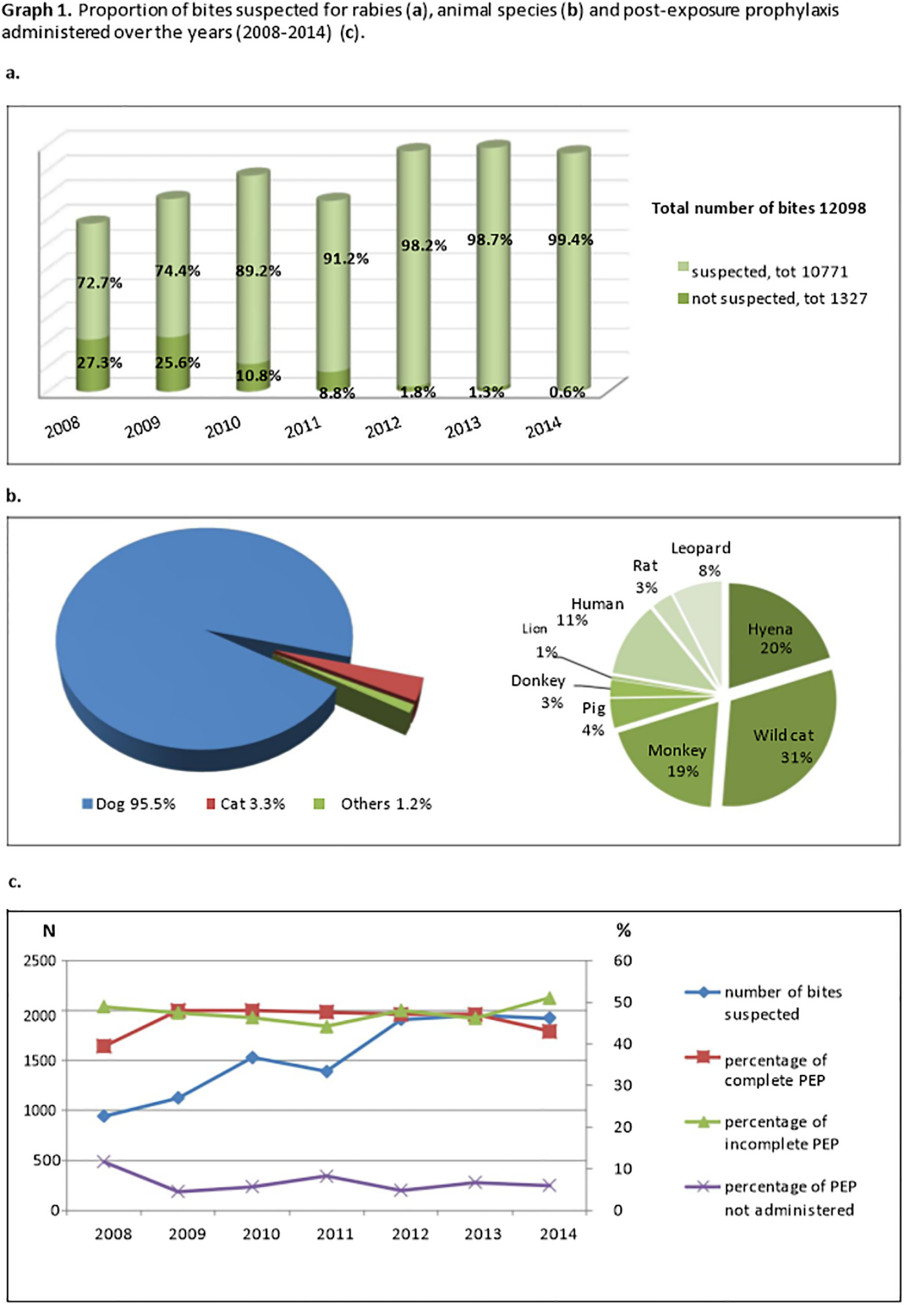
Characteristics of reported suspected rabid animal bites and post-exposure prophylaxis. (a) Proportion of suspected rabid animal bites over total number of bites per year. (b) Animal species responsible for bites. (c) Post-exposure prophylaxis administered between 2008 and 2014.

From 1st January 2008 to 31st December 2014, 14,624 patients attended the DRRH because of animal’s bites. Eighty-three per cent (12,098) individuals came from Dodoma Region. Among them, 89.0% (10,771) were considered exposed to potentially rabid animals based on clinician assessment, giving a mean incidence of 74 bites considered at risk of rabies transmission per 100,000 persons per year. A dataset of all patients coming from Dodoma Region and considered at risk was created and the statistical analysis was conducted on this dataset. Dodoma Municipality and Chamwino District reported the higher number animal bites considered at risk of rabies transmission over the period of study, accounting for 32.0% (3500) and 23.5% (2537) of bites, respectively (Fig 1). The number of bites considered at risk of rabies transmission has been progressively increasing over the 7-years period. The animal responsible for the bites was most frequently a dog (Fig 2).

During the study period, children under 15 years old accounted for 53.0% (5705) of suspected rabid animal bites. Only 61.6% (6632) of the patients attended the clinic within 48 hours after the bite, while 27.0% (2917) referred to DRRH within 7 days and 11.4% (1222) even more than one week after being bitten (Table 1).

**Table 1:** Patients’ characteristics, delay, PEP administration and follow up of suspected rabid animal bites.

	2008	2009	2010	2011	2012	2013	2014	Total
Suspected rabies bites (N)	942	1123	1530	1390	1911	1950	1925	10,771
**Sex (column %)**								
Male	528 (56.1)	648 (57.7)	851 (55.6)	788 (56.7)	1124 (58.8)	1156 (59.3)	1130 (58.7)	6225 (57.8)
Female	407 (43.2)	475 (42.3)	679 (44.4)	599 (43.1)	781 (40.9)	794 (40.7)	795 (41.3)	4530 (42.1)
Not known	7 (0.7)	0	0	3 (0.2)	6 (0.3)	0	0	16 (0.1)
**Age (years old), N (column %)** **Median (IQR) = 13 (24)**								
<15	508 (53.8)	604 (53.8)	837 (54.7)	740 (53.3)	990 (51.8)	1011 (51.8)	1015 (52.7)	5705 (53.0)
15–49	320 (32.7)	399 (35.5)	523 (34.2)	501 (36.0)	694 (36.3)	718 (36.8)	701 (36.4)	3843 (35.7)
≥50	127 (13.5)	120 (10.7)	170 (11.1)	149 (10.7)	227 (11.9)	221 (11.4)	209 (10.9)	1223 (11.3)
**Delay (days), N (column %)**								
≤2	560 (59.5)	655 (58.3)	941 (61.5)	817 (58.8)	1149 (60.1)	1237 (63.4)	1273 (66.2)	6632 (61.6)
3–7	264 (28.0)	310 (27.6)	402 (26.3)	390 (28.0)	537 (28.1)	515 (26.4)	499 (25.9)	2917 (27.0)
>7	118 (12.5)	158 (14.1)	187 (12.2)	183 (13.2)	225 (11.8)	198 (10.2)	153 (7.9)	1222 (11.4)
**Completion of PEP, N (column %)**								
Complete prophylaxis	372 (39.5)	540 (48.0)	735 (48.0)	662 (47.6)	900 (47.1)	918 (47.1)	828 (43.0)	4955 (46.0)
Incomplete prophylaxis	461 (48.9)	533 (47.5)	708 (46.3)	614 (44.2)	920 (48.1)	901 (46.2)	981 (51.0)	5118 (47.5)
No dose received	109 (11.6)	50 (4.5)	87 (5.7)	114 (8.2)	91 (4.8)	131 (6.7)	116 (6.0)	698 (6.5)
**Loss at follow up N (column %)**								
Lost after I dose	166 (36.0)	154 (28.9)	292 (41.2)	207 (33.7)	347 (37.7)	368 (41.1)	395 (41.8)	1929 (38.0)
Lost after II dose	295 (64.0)	379 (71.1)	416 (58.8)	407 (66.3)	573 (62.3)	527 (58.9)	549 (58.2)	3146 (62.0)

OR = odds ratio

At univariate and multivariate analysis, adults were more likely to delay the access to the hospital compared to children (p<0.001) as well as people living in rural area compared to those living in urban area (p<0.001). Similarly, during rainy season people were more likely to delay the access to care (p = 0.03). Gender was not significantly associated with delay (Table 2).

**Table 2:** Predictors associated with delay to health care access among people with suspected rabid animal bites in Dodoma, Tanzania (10,771).

Characteristics	Univariate OR 95% CI	p-value	Multivariate OR 95% CI	p-value
**Age (years old)**		<0.0001		<0.0001***
<15	Reference		Reference	
≥ 15	1.30 (1.22–1.40)		1.41 (1.32–1.52)	
**District**		<0.0001		<0.0001***
Urban	Reference		Reference	
Rural	2.78 (2.55–3.03)		3.01 (2.77–3.26)	
**Season**		0.08		0.03***
Rainy season	1.07 (0.99–1.15)		1.08 (1.01–1.16)	
Dry season	Reference		Reference	
**Sex**		0.38		
Male	0.97 (0.89–1.04)			
Female	Reference			

OR = odds ratio

Overall, 46.0% (5955) of the total number of individuals exposed to potentially rabid animals completed the PEP course, while 6.5% (698) did not receive any dose. Regarding the overall completion of ARV, children and males were more likely to receive all the scheduled doses (p<0.001). Bites occurring during the rainy season were inversely associated with completion of PEP (p<0.001) (Table 3).

**Table 3:** Predictors associated with completion of PEP among the total number of people with suspected rabid animal bites from 2008 to 2014 in Dodoma Region (10,771).

Characteristics	Univariate OR 95% CI	p-value	Multivariate OR 95% CI	p-value
**Age (years old)**		0.0002		< .0001***
<15	1.15 (1.07–1.25)		1.26 (1.18–1.35)	
≥ 15	Reference		Reference	
**District**		0.002		0.0738
Urban	1.14 (1.05–1.24)		0.94 (0.88–1.01)	
Rural	Reference		Reference	
**Season**		0.006		< .0001***
Rainy season	0.90 (0.83–0.97)		0.87 (0.81–0.93)	
Dry season	Reference		Reference	
**Sex**		0.0003		< .0001***
Male	1.13 (1.06–1.21)		1.16 (1.08–1.24)	
Female	Reference		Reference	

OR = odds ratio

Living in rural area was statistically associated with loss to follow up after the first dose (p<0.001) or after the second dose (p<0.001) Females were more likely to be lost after the first dose (p = 0.006). Both age and season were not related to loss to follow up.

## Discussion

During the study period, DRRH was the only health care facility in Dodoma Region identified as reference centre for rabies prevention and control, serving all the districts within the regional borders and nearby regions. Therefore, the data presented in this study largely reflect the trend of exposure to potentially rabid animals and ARV administration in the entire region from 2008 to 2014. During the same period, 48 suspected rabies deaths occurred at DRRH, although post-mortem confirmation was not available (data retrieved from hospital’s records).

The rate of suspected rabid animal bites has been rising over the years (Fig 2a). A possible explanation of this could be a more accurate adoption of the WHO approach—based on epidemiology, characteristics of exposure and clinical criteria—to assess the risk of developing rabies after being bitten by an animal [8]. Still, the reporting tools used at DRRH are lacking important information on the animal behaviour, on the reasons for considering the bite as potentially at risk of rabies transmission, on the availability of PEP at the risk assessment visit, etc. The “six-step method” proposed by Tepsumethanon *et al*., based on the dog’s age and state of health, on the symptoms’ onset and evolution, on the progress of the illness during the last 3–5 days, on the presence of the “sign of circling” and of other behavioural signs, demonstrated 90.2% sensitivity, 96.2% specificity and 94.6% accuracy for the presumptive diagnosis of rabies in living dogs [13]. This method could strengthen the assessment of suspected rabid animal bites and prioritize ARV and RIG administration, if available.

The delay in hospital attendance for PEP administration decreased year-by-year while the proportion of patients attending the DRRH within two days after being bitten increased by 7.0% (Table 1). These data suggest that there is a higher awareness of the potential risk associated with animal bites and of the importance of attending the hospital shortly after being bitten. Anyway, the overall percentage of patients that attended the referral centre more than 7 days after the bite is still too high (11.4%).

In agreement with previous report [4,14], our survey shows that children under 15 are the most exposed to rabies risk. In addition, our data show that adults were more likely to delay health care access (Table 2) and to fail to complete PEP (Table 3). Children’s lower rates of delay to health care access and children’s higher completion of the scheduled vaccination may reflect a greater attention paid to child health.

Our results seem to be affected by the centralization of rabies services. We found that residence in a rural area was a predictor of delay to health care access and of failure to complete the PEP. Also, being bitten during rainy season was significantly associated to delayed and incomplete PEP, which may reflect greater difficulties in travelling to DRRH from a rural area during that period [15]. Interestingly, despite the number of bites considered at risk of rabies transmission increased progressively, the percentage of complete ARV did not vary (Fig 2c), with only less than half of the patients completing the prophylaxis. Until 2014, the national policy provided free PEP to people exposed to the risk of developing rabies. Nonetheless, drug shortages are frequent in LMICs health care facilities and patients may not be able to sustain the vaccine costs, thus deciding to go back to traditional practices or to stop the treatment course. A 4-dose-full-course of rabies PEP would cost 100,000 Tanzanian Shillings (44 USD, approximately) while, based on 2012 Household Budget Survey, more of a quarter of the Tanzanian population fall below the basic needs poverty line (36,482 Tanzanian Shillings per adult per month, equivalent to approximately 16 USD) [16]. Several strategies have been put in place in order to bridge this gap in many LMICs [17]. Recently, rabies prevention and control in Dodoma Region has been decentralized, allowing peripheral health centres to provide rabies PEP without referring the patients to DRRH. The availability of ARV, the maintenance of the cold chain and the affordability of vaccination for people living near the poverty line are still major concerns to be addressed at a national and international level [15].

Females had the lowest rates of full vaccinations, probably because of the economic dependence on their families for transportation and for PEP cost. This finding reveals that the affordability of vaccine supplies is currently an important challenge to address since it may also contribute to social and gender inequalities.

Recent reports show that a decrease in human rabies cases is possible through a combination of interventions such as dog vaccination, improvement of access to PEP, and implementation of the surveillance and reporting system [17,18]. This is confirmed by the progresses achieved in Philippines, South Africa and in five regions in south-east Tanzania [19]. During the study period, Dodoma region was not involved in any dog immunization campaign, so this aspect of rabies prevention and control needs to be implemented.

According to the latest WHO guidelines, PEP can be administered both through the intramuscular and the intradermal route [20]. The intradermal regimen requires a reduced volume of vaccine thus reducing costs by 60–80% [20]. This option could be particularly suitable in areas with vaccine shortage, low-income settings or high-flow clinics [20]. Given an average annual incidence of 1538 patients with suspected rabid animal bites requiring a full course of PEP, we estimate that the cost saving switching from intramuscular to intradermal regimen during the study period would have been around 40,000 USD per year [21].

The present study has several limitations. First of all it has been conducted retrospectively, meaning that it was not possible to verify the appropriateness of the risk assessment. For the same reason, the authors could not speculate on the clinician’s decision on PEP administration, since the registers did not report any information about the reasons why the PEP was/was not recommended.

## Conclusions

We report data illustrating the challenges related to rabies prevention and control in a tertiary hospital in Tanzania. In particular, our findings indicate that overcoming the economic barriers to post-exposure vaccination is one of the main challenges to be addressed to achieve the goal of rabies elimination. Dog vaccination may also play a pivotal role in rabies prevention [22]. Surveillance and reporting need to be more detailed, with a precise description of the exposure and of the criteria for recommending PEP. Switching from intramuscular to intradermal rabies vaccination should be considered as an effective option to reduce rabies prevention cost in the national health system.

## Supporting information

S1 Dataset Patients’ data collection excel file. Sheet 1: 10,771 patients included in the study (see Materials and methods); sheet 2: 3853 patients excluded; sheet 3: dataset variables legend.
(XLS)
